# Evidence pointing toward invalidity of the SF-8 physical and mental scales: a fusion validity assessment

**DOI:** 10.1186/s12874-024-02387-z

**Published:** 2024-11-11

**Authors:** Leslie A. Hayduk, Matthias Hoben, Carole Estabrooks

**Affiliations:** 1 Department of Sociology, University of Alberta, Edmonton, AB T6G 2H4 Canada; 2 School of Health Policy and Management, Faculty of Health, York University, Toronto, ON M3J 1P3 Canada; 3 Faculty of Nursing, University of Alberta, Edmonton, AB T6G 1C9 Canada

**Keywords:** SF-8, Scale, Validity, Fusion validity, Structural equation model

## Abstract

**Background:**

The SF-8™ Short Form Health Survey creates physical and mental health scale scores from responses to eight survey questions. These widely used scales demonstrate reasonable reliablity, and some forms of validity but have not been assessed for fusion validity. We assess the fusion validity of the SF-8 physical and mental health scales, and provide comments assisting fusion validity assessment of other scales.

**Methods:**

Checking the fusion validity of a scale requires including the scale and its constituent indicators in a structural equation model that has at least one variable causally downstream from the scale. We assessed fusion validity of the SF-8 physical and mental health scales in the context of work-related variables for care aides working in Canadian long-term care homes. Variables causally downstream from physical and mental health, such as work burnout, permit checking whether the SF-8 indicator items fuse to form cogent physical and mental scales, irrespective of whether those indicators share common-factor foundations.

**Results:**

We found that the SF-8 physical and mental health scales did not function appropriately. The scales inappropriately claimed effects for several items that had no effects and provided biased estimates of other effects. These deficiencies seem grounded in the scales’ developmental history, which implicitly bolstered selection of some causally ambiguous items and paid insufficient attention to component factor model testing.

**Conclusion:**

Our observations of causal incongruities question whether the SF-8 can provide valid assessments of physical and mental health. However, it would be imprudent to discontinue SF-8 use on the basis of a single study suggesting invalidity. This uncomfortable conclusion can be rechecked by re-analyzing data from any project that employed the SF-8 and recorded even one causal consequence of physical or mental health. The power of fusion validity assessment comes from connecting the recorded consequences simultaneously to both the scale and the items from which that scale is calculated.

**Supplementary Information:**

The online version contains supplementary material available at 10.1186/s12874-024-02387-z.

## Background: SF-8 History

Scores on the SF-8™ [1] physical and mental health scales are obtained by weighting responses to eight questions. The eight indicator items are a slightly modified subset of 36 items from the Medical Outcomes Study, which in turn was adapted from longer scales in use for decades [2–4]. Ware and colleagues made each item correspond to one of eight health domains, concepts, or dimensions [1, 2]. Reliability of the SF-8 has been checked and component factor analyses run [1]. The importance of physical and mental health, and the substantial history of scale development, prompted translation of the SF-8 items into more than 30 languages [5].

Focusing the items on eight defined areas provides laudable clarity. However, when the SF-36 and SF-8 were developed it was not commonly recognized that principle components factor models assume that the items originate exclusively in latent common causes of the items [6]. For example, a principle component analysis containing an item referring to pain and an item referring to activity limitations claims that the correlation between pain and limitations results from both these items responding exclusively to a latent common cause, rather than the pain producing activity limitation. Investigating possible causal connections among 36 items was not practical, but might have been manageable with eight items. Unfortunately, instead of altering the indicator wordings to correspond to actual causal interconnections, the SF-8 team adjusted item wordings and weightings to force the eight items to correspond to the latent common-cause components previously estimated for the 36 items [1, 3].

Recent factor analyses suggest that – based on χ^2^ tests of model fit – one, two, three, and four constrained factors (including bi-factor models with two and three dimensions in addition to a general factor) are inconsistent with SF-8 data [7, 8]. However, these data inconsistencies have been downplayed by favoring fit indices and discounting the χ^2^ tests of model fit [9]. Differing numbers of underlying causal factors is troubling because it introduces uncertainty about how many things are being measured. If theory claims two factors underlie the items, finding one, three, or four factors points toward invalidity because the correspondence between the measurements and theoretical understanding has faltered. This challenges whether the predictive capabilities of the SF-8 scales constitutes predictive validity because which, or even how many, underlying latents might be operative in any specific context becomes unknown. Additional testing of theoretical claims to common common-factor causes could be introduced by modeling anticipated causes or effects of the two theorized factors [10]. This style of testing would increase the power to detect whether more factors are required than the two claimed by the SF-8.

But evidence reporting misspecification of the items’ causal sources would be insufficient to fully invalidate the SF-8 scales because a different style of theoretical understanding of the scales remains possible. Nothing prevents independently produced items, or even directly causally interconnected items, from combining or fusing to form a unitary causal scale. For example, employment income, game winnings, investment income, and theft have distinct causal foundations, yet could all increase a valid “scale of available funds” possessing clear causal consequences. Similarly, even multiple mingled causal foundations for the SF-8 items would not necessarily preclude items from combining to report on mental and physical states possessing relevant causal capacities. Abandoning the traditional common-cause style of theorizing might be awkward, but tolerable if it recovered SF-8 scale validity. Unfortunately, attempts to disentangle the scale and item effects by including them as predictors in a regression predicting any appropriate dependent variable are derailed by the collinearity (statistical redundancy) between the items and scales.

These observations limit what might be usefully undertaken to further assess the validity of the SF-8. There are enough failing factor models to render additional factor investigations redundant. An additional predictive-validity study would be useful for whatever was being predicted but this would not contribute much to assessing SF-8 validity because successful prediction would leave undetermined whether the predictive capability originates from two valid underlying latents, or from an unknown number of confounded latent variables. And multiple regression attempts to simultaneously assess the effects of any scale and its constituent items are stymied by collinearity among the predictors. Fortunately there remains one additional way to investigate whether SF-8 scales fuse to form valid causally operative scales, whether or not they originate in two underlying latent causes.

Hayduk, Estabrooks, and Hoben [11] demonstrated a structural equation modeling setup that is similar to regression but which circumvents the collinearity issue that blocks regression. Their fusion validity analysis evades the collinearity problem by modeling the scale as a latent variable produced exactly the same way the researcher calculates scale scores by weighting item responses. The procedure weights, fuses, or combines the items’ values using the weightings required by the scale, and the validity assessment arises from checking whether the items and resultant scale act theoretically-appropriately with respect to non-scale variables. The Hayduk, Estabrooks, and Hoben fusion validity assessment considered only a single scale, so it initially remained unknown whether collinearity could similarly be circumvented with two scales – the SF-8 physical and mental health scales – when both scales were assessed simultaneously and based on the same small set of indicator items.

This provided hope that the statistical complications could be overcome but some cognitive tangles remained. Scales are supposed to simplify matters by replacing the multiple items, so why re-complicate matters by reintroducing the presumably now-unnecessary items? The reason is that modeling a scale along with its items permits checking whether the scale actually validly simplifies matters by rendering the indicators redundant, or whether the researcher’s causal actions in producing a scale merely cloak misunderstanding. Just as a causally ineffective variable can be a significant predictor in regression if it is confounded with a causally effective variable, a scale can be predictive despite misrepresenting the items. A model containing both the items and the SF-8 scales permits checking whether the items actually do fuse to form physical and mental dimensions capable of impacting causally-downstream variables, and whether the scales fully and appropriately represent the causal capacities of the eight items. The additional power of a fusion validity assessment comes from simultaneously examining the effects of the scale while controlling for the items, and the effects of the items while controlling for the scale. Modeling a scale along with its constituent items provides a stronger validity assessment because it provides a fuller picture of the theoretical claims required to validate the scale than is possible by employing just the items or just the scale.

Below we describe our data, fusion validity models, and results – results revealing substantial problems in the SF-8. Subsequently we consider matters relevant to checking fusion validity of scales more generally, such as: Why was scale invalidity not detected sooner? Which standard operating procedures failed to detect the problems? Why did standard procedures falter? Does scale failure have deeper consequences for psychometrics more generally? What can be usefully done if a scale fails? We address these concerns in the context of the SF-8, while also striving to assist fusion validity assessment of additional scales.

## Methods

### Setting and Sample

This analysis used cross-sectional data collected from care aides in an ongoing cohort study embedded within the Translating Research in Elder Care (TREC) program of research, an applied program of health services research [12]. We used data from TREC’s wave 4 of data collection (May 1, 2017 to December 19, 2017). TREC long-term care homes are a representative sample of urban homes, randomly selected from lists stratified by (a) health region (Calgary and Edmonton Health Zones in Alberta, Fraser and Interior Health Regions in British Columbia, Winnipeg Health Region in Manitoba), (b) size (small: <80 beds, medium: 80–120 beds, large: >120 beds), and (c) ownership model (private not-for-profit, voluntary not-for-profit, private for-profit). Trained data collectors completed structured, computer-assisted interviews with 4,158 care aides in 94 long-term care homes, using validated surveys [12]. We excluded 493 care aides who worked primarily night shifts, expecting their responses to systematically differ from care aides working primarily morning or evening shifts.

### Measures and Models

SF-8 data were gathered using the item wordings from Ware and colleagues [1:128], with a four-week reference period. To avoid infringing on our license agreement, Table 1 paraphrases the SF-8 items with wordings relevant to our discussion and employing new variable designations: Rated Health (**RH**), Physical Limits Activities (**PLA**), Physical Limits Work (**PLW**), Bodily Pain (**BP**), Energy (**EN**), Physical Emotional Limits Social (**PELS**), Bothered by Emotions (**BE**), Personal Emotional Limits Work (**PELW**). Responses to the SF-8 items were initially recorded by TREC using integer designations but were later recoded to correspond to the SF-8 response values [1:18], where higher values correspond to better health. We used the recoded values in SAS^®^ 9.4 to calculate the means, variances, and covariances for these and the other modeled variables (Table 1). The physical and mental scale values were calculated using the SF-8 weights from Ware and colleagues [1:19]. Most aides were female (90%) and 65% had English as a second or additional language. They were heterogeneous in age, with most distributed throughout the range of 25 to 65 years (Table 1). No directly comparable reference-group values for the SF-8 items and scales are available, but our participants seem to be slightly more healthy and energetic but less bothered by emotions than the general US comparison group reported in the SF-8 manual [1:34]. The general Canadian population scored slightly higher than the US population on the SF-36 [13] but differences by sex, age, and ethnic groupings render comparisons difficult.

**Table 1:** Modeled variables

Variables and Their Designations	Mean^k^	Standard deviation	Variance	Assigned percent measurement error
RH How would you rate your overall health?^a^	**50.04**	**7.06**	**49.84**	**5**
PLA Extent physical health limits activities like stairs.^a^	**48.06**	**7.26**	**52.66**	**5**
PLW Extent physical health limits work, home and away.^a^	**48.33**	**7.26**	**52.64**	**5**
BP Extent of bodily pain.^a^	**49.05**	**8.71**	**75.93**	**5**
EN Amount of energy you have.^a^	**55.15**	**7.56**	**57.09**	**5**
PELS Extent physical or emotion limits social activity.^a^	**48.56**	**7.75**	**60.14**	**5**
BE Extent bothered by emotions.^a^	**50.37**	**7.81**	**60.92**	**5**
PELW Extent personal problems or emotions limit work.^a^	**47.76**	**6.00**	**36.02**	**5**
SF-8 Physical scale^b^	**49.10**	**8.07**	**65.09**	**0**
SF-8 Mental scale^b^	**51.86**	**8.54**	**72.94**	**0**
Enough Staff We have enough staff to get the necessary work done.^c^	**3.12**	**1.28**	**1.63**	**5**
Supportive I am a member of a supportive work group.^c^	**4.03**	**0.75**	**0.56**	**4**
Sex ^d^	**1.90**	**0.30**	**0.09**	**3**
Age ^e^	**6.59**	**2.27**	**5.14**	**4**
ESL English as a second language.^f^	**1.65**	**0.48**	**0.23**	**20**
DRAN Disruptive resident actions – nonsexual.^g^	**2.87**	**1.31**	**1.71**	**5**
DRAS Disruptive resident actions – sexual^h^	**0.38**	**0.67**	**0.45**	**5**
Tired AM I feel tired when I get up in the morning and have to face another day on the job.^i^	**2.89**	**2.07**	**4.27**	**2**
Rushed^j^	**3.00**	**2.77**	**7.65**	**7**
Burntout I feel burned out from my work.^i^	**2.40**	**2.01**	**4.04**	**4**
Look Forward I look forward to going to work.^i^	**5.33**	**1.21**	**1.47**	**4**

^a^ this variable’s values are coded according to Ware, Kosinski, Dewey and Gandeck (2001:16–18)

^b^ this variable’s values are calculated using the weightings from Ware, Kosinski, Dewey and Gandeck (2001:19)

^c^ this variable’s values are coded 1 = strongly disagree, 2 = disagree, 3 = neither agree nor disagree, 4 = agree, 5 = strongly agree

^d^ this variable’s values are coded 1 = male, 2 = female

^e^ this variable’s values are coded 1 < 20, 2 = 20–24, 3 = 25–29, 4 = 30–34, 5-35-39, 6 = 40–44, 7 = 45–49, 8 = 50–54, 9 = 55–59, 10 = 60–64, 11 = 65–70, 12 > 70

^f^ this variable’s values are coded 1 = yes, 2 = no

^g^ this variable’s values are a count, of four possible, kinds of resident actions the aide reported having experience in their last 5 work shifts (yelling or screaming; verbal threats; hurtful remarks or behaviors; being spit on, bitten hit, pushed or pinched)

^h^ this variable’s values are a count, of two possible, kinds of resident actions the aide reported having experience in their last 5 work shifts (sexual touching; repeated and unwanted questions or remarks of a sexual nature)

^i^ this variable’s values are coded: 0 = never, 1 = a few times a year or less, 2 = once a month or less, 3 = a few times a month, 4 = once a week, 5 = a few times a week, 6 = daily

^j^ this variable’s values are the number of tasks the aide reported having been rushes to preform to a maximum of seven

^k^ N ranges from 3660 to 3665

The variables causally downstream from the SF-8 in our models were collected using the validated TREC care aide survey, extensively used in previous studies [14–17], with details reported elsewhere [12]. These variables depict the work-life experiences of care aides: whether they begin their work-day feeling tired, how rushed they feel at work, if they feel burned out, and whether they look forward to their next work shift. All our models causally interconnect these work variables in the same way. Aides who feel tired when beginning their work-day are presumed to: feel rushed at work (when tired, the world seems to move faster), feel burned out (feeling tired before one begins), and be less likely to look forward to their next shift (not being ready for it). Being rushed is assumed to contribute directly to burnout, and feeling rushed or burned out is assumed to make the next shift seem less appealing.

All our models also contain the same control variables from the TREC care aide survey, which include: organizational features that can contribute to downstream variables (having enough/insufficient staff, having a supportive/unsupportive work team); situations that complicate or hinder a care aide’s work tasks (sexual or non-sexual disruptive resident actions); and personal characteristics (sex, age, English as a second or additional language – potentially connecting to communication skills or racialized resident responses). In all our models, control variables and connections among care aides’ work-life variables function nearly as expected. Placement of effects and absences-of-effects connected to these variables were not altered from their original specification (by LH) before initial model estimation. This consistency maintains a focus on how the SF-8 scales and indicator items function in our models.

**Fig. 1:**
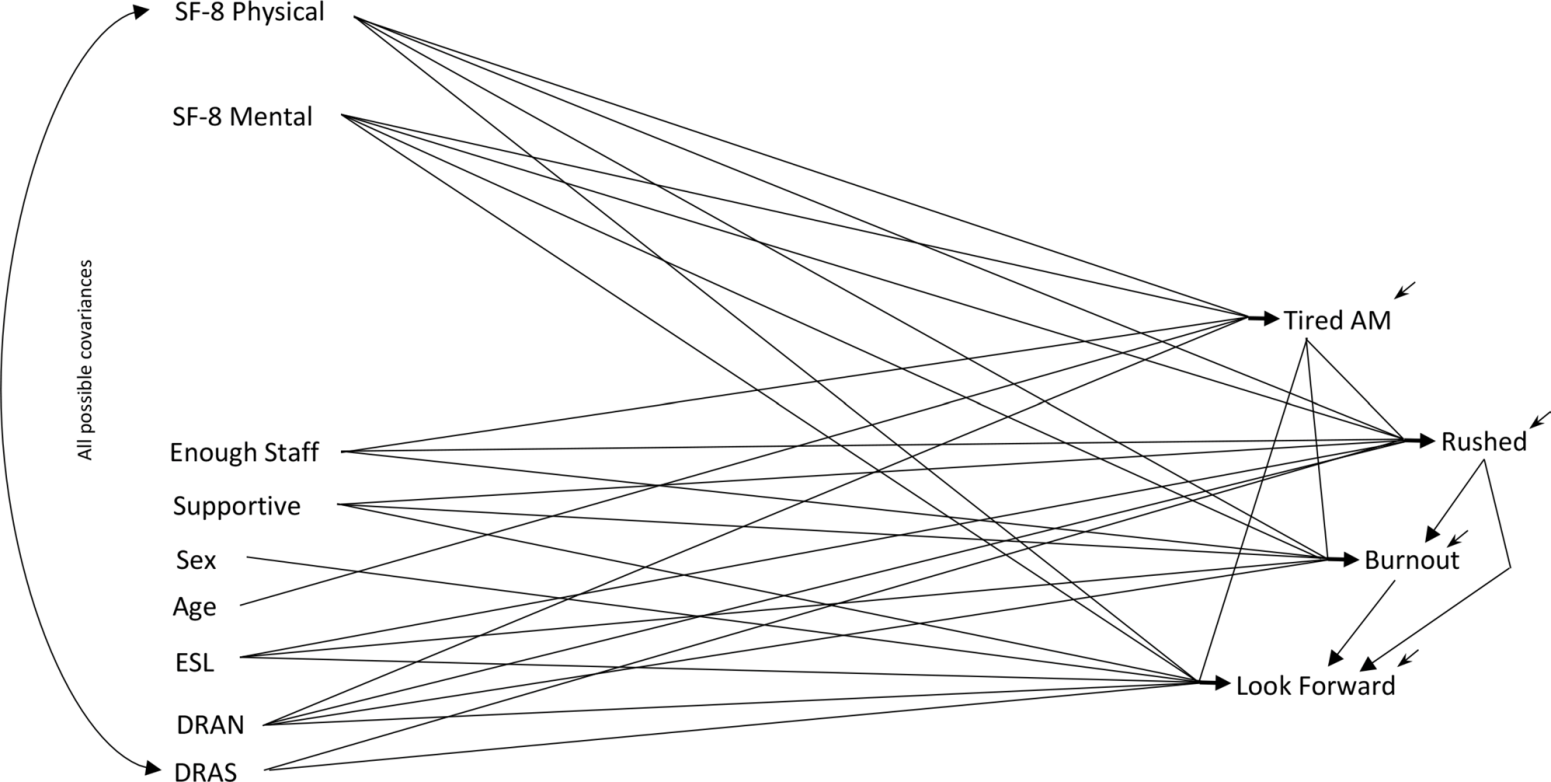
The Scale-Only Model (*The named variables are described in the text*)

**Fig. 2:**
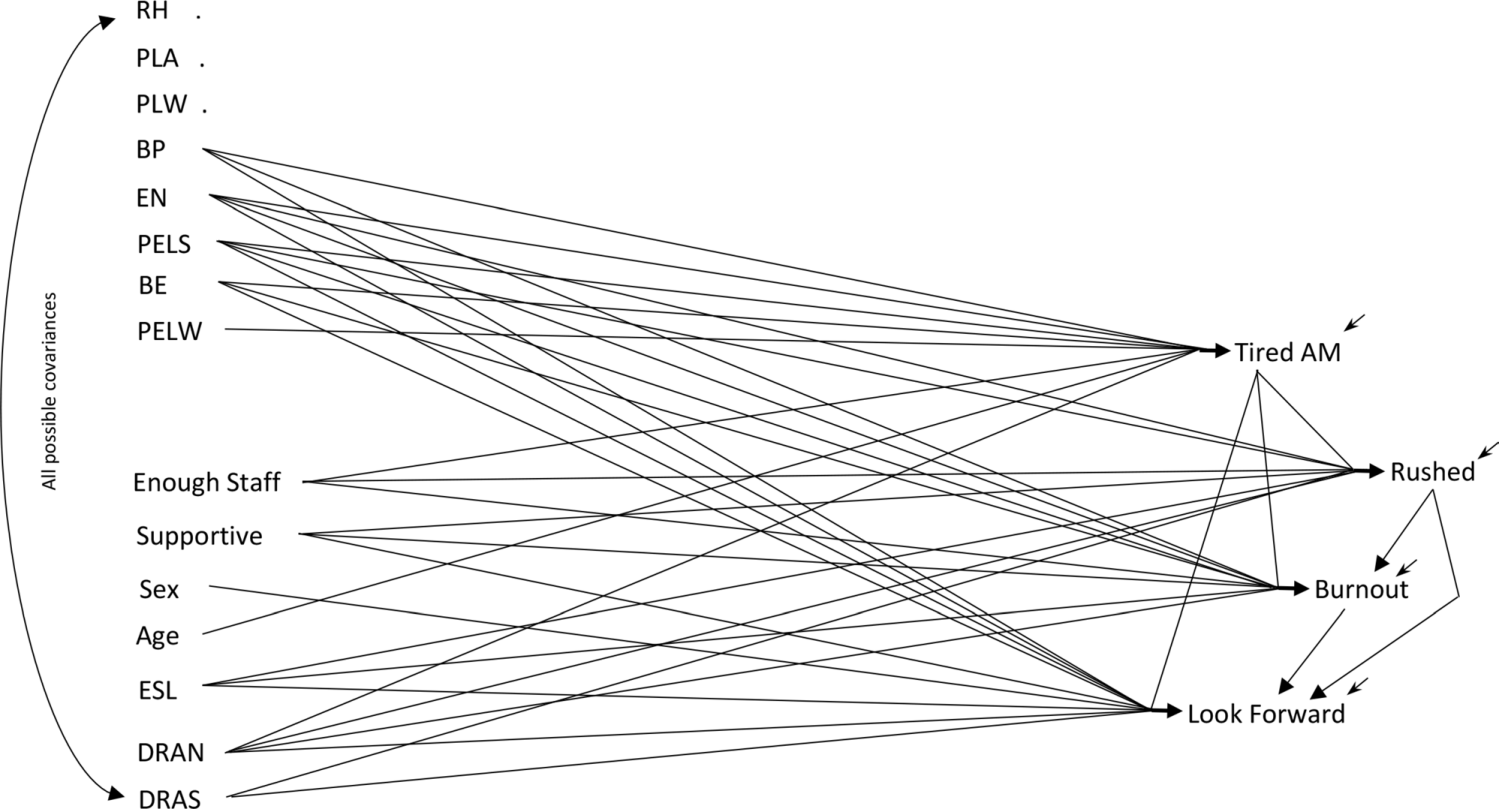
The Item-Only Model (*The named variables are described in the text. 5% measurement error variance was provided for the SF-8 items. The first three of the SF-8 items have no effects*)

**Fig. 3:**
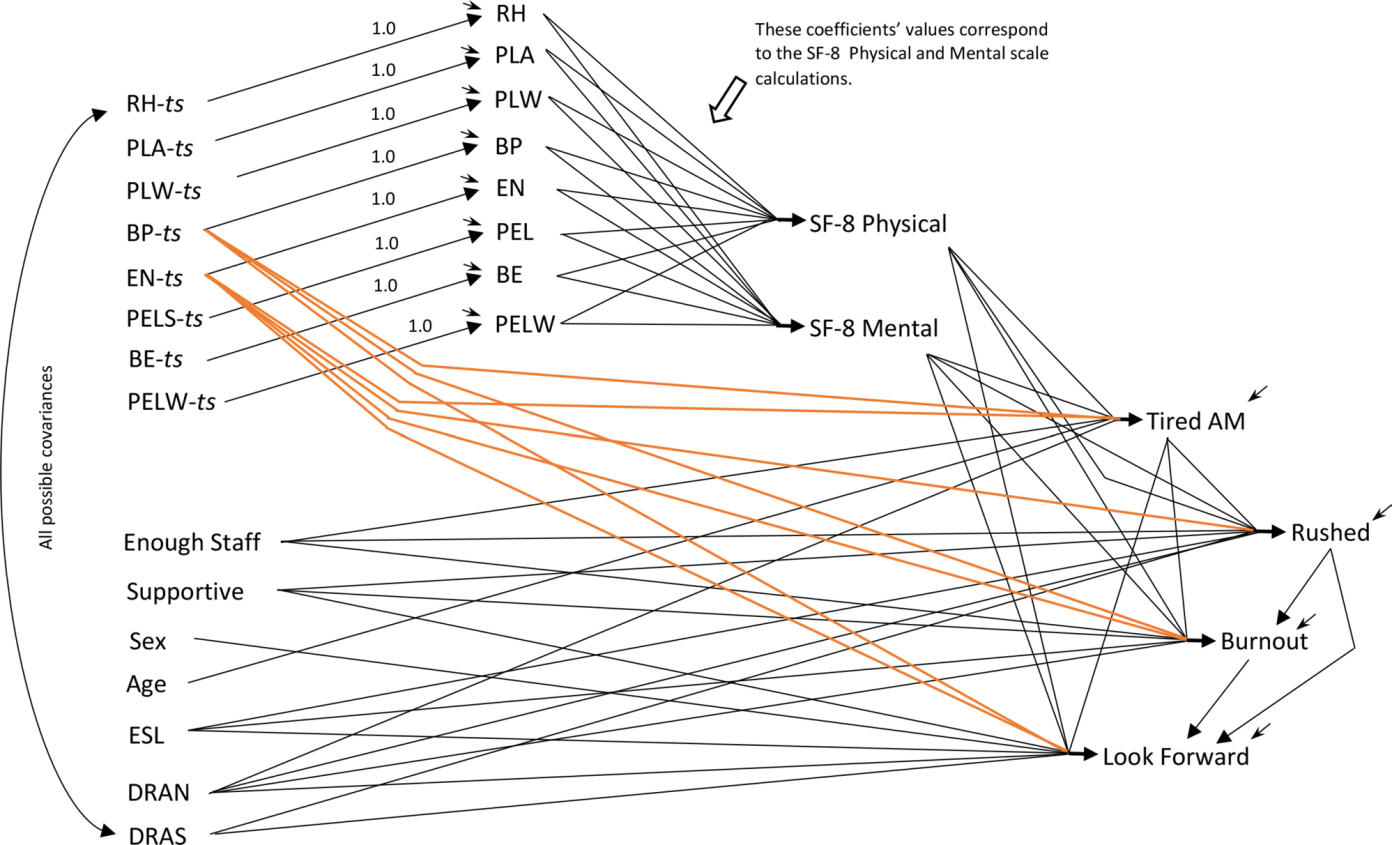
The Scale-and-Item Model (*The named variables are described in the text. ts indicates a 5% measurement error variance adjustment to the corresponding indicator item provided this “true score” latent variable*)

The s*cale-only* model (Fig. 1) includes the SF-8 physical and mental scales as causes of the work-life variables. The *item-only* model (Fig. 2) uses the eight SF-8 indicator items, instead of the scales, as causes of the work-life items. The s*cale-and-item* model (Fig. 3) is an extension of the basic fusion validity model, incorporating both the indicator items and the SF-8 scales as possible causes of care aides’ work-life experiences. This sequence of *scale-only*, *item-only*, *scale-and-item* models clarifies the logical structure of the fusion validity assessment for the SF-8, though the models were developed in a different sequence. The *scale-and-item* model (Fig. 3) was developed first, then the models in Figs. 1 and 2, for reasons explained below.

## Results

### The Scale-Only Model

The scale-only model permits potential effects of the SF-8 physical and mental scales on each downstream variable, because better physical and mental health might result in: less morning tiredness, fewer feelings of being rushed at work, less burnout at work, and more positive outlook toward the next work shift. Maximum likelihood estimates for this model, obtained using Linear Structural Relations (LISREL) [18], appear in the top row in each set of triplicate rows of Table 2. The corresponding covariance matrix and other modeling details are reported in Appendix A. This model fits the covariance data nicely (χ^2^ = 7.23, *df* = 11, *p* = .78). Most effects are somewhat weak though highly statistically significant, given the substantial sample size. All eight of the possible effects of physical and mental scales on the downstream variables are statistically significant and have the proper sign. Better physical health (as a scale) and better mental health (as a scale) seem to reduce tiredness, reduce feeling rushed, reduce burnout, and increase looking forward to the next shift. All would seem well by modeling the SF-8 physical and mental scales in this typical way. Problems in this scale-based perspective remain hidden until we consider our other models.

**Table 2:**
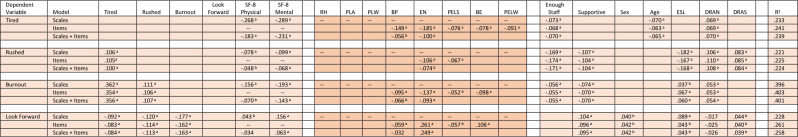
Standardized Direct Effects

Estimates for the models in Figs. 1 and 2, and 3 appear as rows labeled Scales, Items, and Scales + Items respectively. The effects leading to, and defining, the SF-8 Physical and Mental scales in the scale-and-item model correspond to the values in Ware, Kosinski, Dewey and Gandeck (2001:19) but are not shown, and the standardized version of the 1.0 effects of the true-score on the SF-8 items in the scale-and-item model (Fig. 3) are also not shown. The SF-8 items appear in the darker middle portion of this table, and all the variables are described in the text and Table 1

-- the corresponding coefficient is not in this model because the corresponding causal variable is not part of the model

^a^ the corresponding unstandardized estimate exceeds 3 standard errors

^**b**^ the corresponding unstandardized estimate exceeds 2 standard errors

### The Item-Only Model

The indicator-item-only model (Fig. 2) replaces the SF-8 physical and mental scales with the eight items from which the scales are constructed. The maximum likelihood estimates for this model appear in the second/middle row in each set of triplicate rows in Table 2. This model also fits the covariance data (χ^2^ = 23.60, *df* = 28, *p* = .70) with weak but significant effects. This ‘purely item’ perspective on the care aides’ work world results in three SF-8 items displaying no effects. One item has only a single effect and the remaining four items display three or all four of their possible effects on the downstream variables. We need our last model’s results, and the models’ development histories, to appreciate these estimates.

### The Scale-and-Item Model

Figure 3 depicts an augmented fusion validity model containing the SF-8 scales and the eight indicator items from which the scales are constructed. The physical and mental scales are created as latent variables whose causal foundations correspond exactly to the causal actions undertaken in calculating the scales’ values from the SF-8 indicators. Each item is provided exactly the same fixed effect magnitude leading to the physical and mental scales that would be employed to calculate scale scores as required by Ware and colleagues [1:19], though our SF-8 license preludes presenting the precise values.

The effect estimates in this model appear in the third/bottom row in each set of triplicate rows in Table 2. This model also fits the covariance data (χ^2^ = 24.55, *df* = 28, *p* = .65) with generally weak but significant effects. Degrees of freedom for this model happen to be identical to those for the item-only model, because the eight additional estimated effects of the physical and mental scales happen to correspond to this model having eight fewer estimated direct effects from the items in the item-only model.

The fundamental feature of this model is that the physical and mental latent variables are identical to the scale score variables a researcher calculates using item weightings from the SF-8 manual. Producing the scale latents precisely from the items makes the scales collinear with the items, but this collinearity is not in the modeled data. It is in the latent level of this model, and in a location that does not interfere with estimation.

Implications of these models depend on their development and the data-demanded augmentation of the scale-and-indicator model in Fig. 3.

## Model Development

Because this was the first attempt to check the fusion validity of two scales simultaneously, we began by checking whether such a model could even be estimated. The model would contain the items and model-constructed versions of the SF-8 physical and mental scales, but only the scales would be permitted direct effects on the downstream variables we (LH) selected from the TREC data on healthcare aides. We randomly selected data from 100 aides working day or evening shifts to check whether the candidate variables had sufficient variance and reasonable distributions and would permit estimation of the proposed model structure. The important, and difficult, task was to select causally downstream and control variables constituting a substantive and trustworthy context for evaluating the SF-8 scales. The earliest models failed to fit, but the important observation was that the model converged and provided estimates, signaling it would be possible to check the fusion validity of the two SF-8 scales simultaneously.^1^ The final background step in model development involved using the full set of participant data (not just the random 100-aide checkout data) with proper SF-8 coding for each item and with adjustment for measurement error in each indicator item (Table 1).^2^ From here on we considered, with extra caution, the full data and real models with potential consequences for the real scales.

At that point the *scale-and-item* model was the ***basic fusion validity model***, with effects leading from the physical and mental scales to all downstream variables but no effects leading from any indicator item directly to any downstream variable. This model was strongly inconsistent with the data (χ^2^ = 192.25, *df* = 35, *p* < .0001). This unmistakably points to the inability of the physical and mental scales to encapsulate the effects of the items on the downstream variables – but this model failure should be trusted only if the remainder of the model functions reasonably. It was unknown whether item effects bypassing the scales and leading directly to downstream variables would produce a model that was both substantially reasonable and consistent with the data. Each additional direct item effect would challenge the mental or physical health scale’s ability to appropriately represent the items’ effects. We took care to ensure that the additional effects pointing toward scale inadequacy were data-prompted, not reflections of researcher bias. The severe failure of the fusion validity model to fit the covariance data shouted the inability of the scales to encapsulate the items’ effects. What the data would report about specific items remained to be determined by specific data-prompted scale-bypassing effects.

Table 2; Fig. 3 show that only two indicator items, bodily pain (BP) and energy (EN), required additional effects to nearly all the downstream variables for a well-fitting scale-and-item model. The other six SF-8 items required no effects beyond their indirect effects working through the physical and mental scales on any downstream variable. Note that the mental and physical scale variables in this model remain exactly as required by SF-8 scoring, even as the new item effects bypassing the scales reduced the estimated scales’ effects on the downstream variables (Table 2).

That completed the *basic fusion validity model* (the failing model similar to Fig. 3 but without direct effects from indicator items) and the enhanced or expanded scale-and-item model in Fig. 3. Having confirmed the ability to estimate the fusion validity setup with two scales, we turned to the *scale-only* model (Fig. 1; Table 2). This model contains no items, but employs SF-8 physical and mental scale scores from the TREC data set. The scales are permitted possible effects on all downstream variables, as in the scale-and-item model. This model fit reasonably as reported above, with no data-induced changes to causal connections.

The item-only model (Fig. 2) was the last model estimated and contains all the SF-8 items but not the SF-8 physical and mental scales. Eliminating the scales from the model removes opportunities for indirect effects to lead from the items through a scale to downstream variables. If such indirect effects existed, the item-only model would fail without appropriate additional effects leading directly from the influential items to the downstream variables. Eight indicator items and four downstream variables give a total of 32 possible direct item effects. Seven of these direct effects were already required to attain model fit even when indirect effects through the scales were present in the scale-and-item model. However, the data required inclusion of only eight of the remaining 25 effects. If the SF-8 physical and mental scales really transmitted indirect effects from all the SF-8 indicators to the downstream variables, then all or nearly all of the 25 possible effects would be required to attain model fit – but only 15 of the 32 possible effects were required.^3^

## The Three Models Together

The three models in Table 2; Figs. 1 and 2, and 3 agree that understandable but relatively weak effects connect the downstream variables to one another. Being tired leads directly to aides being rushed and burned out. Being tired, rushed, and burned out each individually contribute to aides being less enthusiastic about their next work shift. The control variables also function appropriately. Insufficient staffing contributes to being rushed, burned out, and tired, while being part of a supportive work group reduces being rushed and burned out. Encountering non-sexual disruptive resident activities increases aides’ feeling of being rushed, burned out, and tired and reduces their looking forward to their next shift. Sexual disruptive resident activities contribute to care aides being rushed but make them slightly more likely to look forward to their next shift. All these features seem agreed upon when estimated using just the SF-8 physical and mental scales, just the items, or the items and scales together. The consistency of these reasonable effect estimates means assessment of the scales is not confounded by disagreements about the context provided by the remainder of the model.

The models differ markedly in how the items and scales function. The scale-only model would report reasonable consistency between the model and scale-data, and that both better physical and mental health decrease care aide tiredness, feeling rushed, and burnout while simultaneously increasing care aide optimism about their next shift. The fusion validity check made possible by including items with the scales convincingly demonstrates that the scales do not convey or transmit the collective effects of the items. The severe failure of the basic fusion validity model (the scale-and-item model with no direct item effects) is clear evidence against the SF-8 physical and mental health scales. This evidence alone does not quite devastate the scales because a fitting model (the scale-and-item model, Fig. 3; Table 2) could permit only bodily pain (BP) and energy (EN) direct scale-bypassing effects on the causally downstream variables. The strongest of these additional effects – higher energy leading to looking forward to the next shift – is so strong that it forces the effect of the physical health scale on aides looking forward to the next shift to become essentially zero (actually insignificantly negative). EN is a major contributor to the SF-8 physical health scale, and its demand for its own strong positive direct effect on looking forward forces the overall physical health scale to compensate by essentially eliminating its effect on looking forward. Attempting to salvage the scales by incorporating separate effects for EN and BP would substantially weaken all the scales’ effects on downstream variables (Table 2). Physical and mental health would become both nearly impotent, and unable to appropriately represent effects of BP and EN.

The bad news for the scales does not end there. The model containing the items but no scales (the item-only model, Fig. 2) challenges the SF-8 scales differently. The columns of Table 2 for rated health (RH), physical limits activities (PLA), and physical limits work (PLW) contain no estimated effects for the scale-only model (the first row of each triplicate row of Table 2) because the scale-only model does not contain the item variables. In the scale-and-item model (third/bottom of each triplicate row), absence of direct effects might arise from these items having indirect effects transmitted through the scales to downstream variables. But the item-only model (second/middle of each triplicate row) contains no scales to transmit indirect effects. So these three of the eight SF-8 items display no effect on the downstream variables, even though as components of the scales these variables were forced to display weak indirect effects on all downstream variables to “accommodate” effects from other items. Only one of the eight indicator items (energy EN) displays effects on the downstream variables parallel to the effects claimed by the scales in the scale-only model. Seven of the eight items show no effects at all, a reversal in sign of an effect, or only some of the effects claimed by the scales.

The identity of the ineffective items further challenges the SF-8 scales. How can care aides’ ratings of their general health (RH) not function in causal-parallel to the physical or mental health scales? RH correlates strongly with the physical (0.94) and mental (0.40) health scales because general health ratings are part of the scales and because RH correlates with other scale items. However, something makes RH function causally differently than the SF-8 health scales. Furthermore, the SF-8 items most closely connected to work (physical limitations on work (PLW) and personal emotional limits on work (PELW)) show almost no connection to the work-related downstream variables. This points to another difficulty with the scales, but appreciating this requires considering methodological details we address now.

### The SF-8 Items

The scale inconsistencies recommend reconsidering the SF-8 indicator items. The individual items also have substantial challenges. Our SF-8 license precludes publishing the SF-8 items, but we can raise some red flags. Several SF-8 items contain the word ‘or,’ which makes responses reflect either this *or* that. If the causal network enveloping ‘*this*’ differs from the causal network for ‘*that*,’ the item cannot display consistent causal foundations and consequences. The ‘or’ items are likely artifacts of attempting to “accurately reproduce the SF-36 physical and mental health summary measures” [1:5] while reducing the number of items. The two summary measures were the principle components spanning the eight domains, and retaining items containing ‘or’ slants the analyses toward similarly requiring two components –one for ‘*this*’ and another for ‘*that.’* Clear causal understanding becomes a casualty.

The items showing the clearest effects in our models – energy (EN) and bodily pain (BP) – do not contain ‘or.’ EN displays the most consistent, reasonably signed, and strongest effects of all items. Its standardized total effect on looking forward to the next shift (the furthest downstream variable in our model) is 0.325, double any other item’s total effect in the item-only model. Employing items with single referents, like EN, does not guarantee items will provide strong effects, but we should expect greater causal clarity than with items of mixed ‘or’ heritage.

Another concern with the SF-8 items centers on the boundary between physical and mental health. Correlation between physical and mental health in our scale-only model is 0.20, matching the very low correlation reported by Ware and colleagues [1: 81], but they also report substantial overlap between factors and a correlation of about 0.51 [1: 86–87]. The precise magnitude of the correlation between mental and physical health is less important than recognizing that current medical understanding coordinates many mental features with physical biochemistry of neural activity [20]. Labeling the SF-8 latent factors as mental and physical followed factor-analytic tradition by labeling factors according to whatever seemed common among the highest-loading items – which implicitly incorporated lay understandings of mental and physical. Removing lay understandings from the SF-8 items would be challenging, but researchers have the responsibility of differentiating between mental and physical as appropriate for their research context.

Similar item and latent-factor boundary concerns are raised by Ware and Sherbourne’s claim that important health concepts were not part of the SF-36 or the SF-8, including “family functioning, sexual functioning, cognitive functioning, and sleep disorders” [2:479]. If someone is tired in the morning because they were awake most of the night, is the morning tiredness part of *mental* health or is it a causal consequence of some *physical* sleep disorder? Is family functioning a part of health (physical or mental) or is this a social consequence of health, or possibly even a cause of physical or mental health? Appropriately placed boundaries between physical health, mental health, and the consequences of health are likely to be study-specific – even item-specific – and potentially at odds with the differentiation between physical and mental implicitly incorporated in the SF-8.

An additional challenge for the SF-8 items is questionable dependence on respondents as causal analysts. Items asking respondents about what limited or constrained their actions, or whether they did something “because of” some specified feature, implicitly depend on the respondent assessing the causal foundations of their actions. This may be reasonable in contexts like pain limiting activities, but it is unreasonable to presume respondents can determine whether a decline in energy (EN) or their general health (RH) is limiting their work activities. Care aides’ EN and RH ratings are substantially correlated in our models (0.48), but statistical control locates energy level as affecting their downstream work-life variables, not whatever underpinned their general health rating. Similar concerns, combined with ‘or’ ambiguity, may explain why the two work-related SF-8 items seem nearly causally disconnected from the downstream work-related variables.

When developing the SF-8, Ware and colleagues “included one item for each of the eight health concepts in the SF-36” [1:10, 79]. Lower correlations between some items were attributed to the ability of items to discriminate between the eight conceptual domains [1:79]. Preserving domain differences implicitly retains domain-specific causal features at odds with the common-factor causal structuring implicit in the scales’ foundation. Our models display clear evidence of divergent item behavior, but the free item covariances in our models permit items to have any number of common causes – one, eight, more or fewer. Fusion validity assessment does not assume a specific number of causes underlying the items and does not depend on causal assumptions about possible connections among items. However, direct causal connections among items, such as pain causing work limitations, do fundamentally challenge the factor-analytic methodology previously used in attempting to validate the SF-8 [1, 6].

### Discussion: The Big Picture

When the 36 items of the Medical Outcomes Study proved too demanding of respondent time, the survey items were progressively reduced until only one item represented each of eight domains considered relevant to physical and mental health. Specified weights combined the eight items into physical and mental health scale scores proffered as appropriate for measuring outcomes of medical trials and other interventions and treatments [1:81–82, 4]. But mathematically combining items to form an internally valid scale requires that the items actually fuse, meld, or coalesce in a way that recovers a corresponding causally operative entity. To check whether the SF-8 physical and mental health scales function causally appropriately, we examined the scales and corresponding items in the context of work-life assessments provided by care aides employed in long-term care homes.

Connections among care aides’ work-world variables themselves are clear and understandable. Specifically, a care aide who is tired in the morning should feel more rushed at work because the world comes at them faster than they can handle. Routinely feeling rushed should in turn lead to burnout and to being less likely to look forward to the next work shift.

The modeled control variables also provide clear and understandable connections. Having enough staff reduces care aide tiredness, feeling rushed, and burnout, while having supportive colleagues reduces feeling rushed and burnout and makes care aides more likely to look forward to their next shift. Disruptive resident actions increase care aides’ workload, contributing to being tired, rushed, and burned out, and reduces looking forward to their next shift. Disruptive actions that care aides viewed as sexual also lead to the care aides feeling more rushed, but incline the care aides toward looking forward to their next shift. Collectively these observations document the care aides’ work world as a reasonable and understandable context for investigating the SF-8 scales.

Permitting the SF-8 physical and mental health scales to influence all the work-world variables provides the *scale-only* model (Fig. 1, and the top of each triplicate-row in Table 2), which fits adequately, displays no obvious problems, and contains significant scale effects of appropriate sign leading to the work variables. These estimates illustrate a style of evidence that could be published as demonstrating the predictive validity of the SF-8 scales because this constitutes a concrete example of the scales functioning in accord with theoretical expectations. This is a comparatively strong type of validity evidence but just as face-validity might be overridden by evidence of predictive invalidity, predictive validity remains open to challenge by stronger evidence. Strong evidence comes from observing that a scale accords with theorized connections, but stronger evidence comes from considering whether both the scale and its constituent items accord with the theorized connections. The collinearity between a scale and its constituent items prevents regression from contributing this stronger style of validity assessment. A fusion validity assessment circumvents this collinearity problem by specifying the scale as a latent variable whose causal foundations correspond exactly to the causal actions undertaken when calculating scale scores from the items.

The extreme failure of the model containing both the SF-8 items and scales (like the scale-and-item model in Fig. 3, but with no direct item effects on downstream work-life variables) unequivocally demonstrates the inability of the scales to encapsulate or adequately represent the items. Results for the physical and mental health scales in the scale-only model (the top rows in Table 2, and in the literature generally) look acceptable and scale-validating, but only because the items are not present in the analyses to demonstrate the scales’ misrepresentation of the items. To obtain a fitting scale-and-item model, the data demanded insertion of seven effects bypassing the scales and leading directly from specific SF-8 items to the downstream variables (Fig. 3). These seven data features each explicitly confront the physical and mental scales. They collectively constitute compelling evidence that the scales do not provide valid representation or understanding of the causal connections for the eight indicator items.

SF-8 scale supporters might retort that even if a few items showed unique effects, the scales might still encapsulate important, possibly essential, features of the full set of items. This view, however, requires acknowledging the substantial weakening of the scales’ already modest estimated effects, and that future valid scale use would require retaining and controlling for the energy (EN) and bodily pain (BP) items to adjust for their scale-bypassing effects. But even these concessions would be insufficient.

The *item-only* model (Fig. 2) demonstrates that three of the eight indicator items display no effects whatsoever. It becomes inconsistent, even illogical, to include these items in scales that supposedly display effects. In short, the radically different functioning of the items (some showing no effects, some showing multiple effects, and some mixed effects) renders pursuit of collective effects futile and suggests that no narrowed conceptualization of physical or mental health is likely to span the SF-8 items.

Ware and colleagues thought that “By relying on only one item to measure each of its eight domains, the SF-8 represents the ultimate phase in the evolution of “static” short form (SF) assessments – the measurement of all SF-36 health concepts with only eight questionnaire items” [1:116]. We think the post-ultimate phase discards the implicit common-cause thinking underlying the SF-8 scales, replacing it with fewer items targeting specific domains. The ability to adjust for measurement error in even single indicators [21] makes it possible to employ fewer indicators focused on the precise health domains most appropriate for assessing a specific intervention or treatment.

### Discussion: Fusion Validity

A fusion validity assessment checks whether items included in a scale fuse or combine to provide scale values corresponding to a causally imbued entity. The procedure creates a latent variable having values identical to the values that would appear in the data set when the researcher applies the required scale weightings to the scaled items. The item weights mathematically combine the items’ values into unitary numerical entities, but it is dubious to claim the resulting scale “measures” something unless that scales’ numerical values function in causal accord with a parallel worldly entity constituting the thing supposedly measured. Seeking evidence of whether the latent scale functions in accord with a worldly causal entity requires that there be at least one variable causally downstream from the scale. More downstream variables are preferable – presuming sufficient understanding undergirds appropriately modeling of those downstream variables. The requirement for appropriate causally downstream variables may be difficult to satisfy, and may require context-appropriate control variables, but demonstrating substantive understanding of how a scale should connect to downstream variables contributes importantly to establishing the assessment as a validity assessment, not a reliability assessment. In this respect, fusion validity parallels the foundational logic of predictive validity. But fusion validity extends beyond predictive validity because the simultaneous inclusion of the items permits checking whether a scale’s predictive capacity originates in some real corresponding feature, or if a scale’s predictive ability originates in mere statistical confounding of the scale with items having real but dissimilar and/or diverse causal capabilities.

Fusion validity assessment is agnostic about the items’ sources. It focuses on items’ indirect impacts on variables further downstream as a consequence of item contributions to the scale. This means confirmatory factor models and fusion validity models investigate different yet complementary causal features. Factor model testing examines the causes of items; fusion validity testing addresses effects of items. The corresponding model tests are complementary, not alternatives. Either, both, or neither of the model tests might be failed.

This article demonstrates that a fusion validity assessment is possible even if two different scales are simultaneously created from the same set of items, and even with only a rather small number of items per scale.

### Discussion: SF-8

An extensive developmental history supported the SF-8 scales from a variety of perspectives [1], but insufficient attention to causal structuring seems to have nonetheless resulted in these scales exceeding their best-before date. The SF-8 scales’ problems seem grounded in the SF-8 indicator items. The item problems seem to trace back to attempting to make the SF-8 scales match two component factors previously obtained using 36 original items. Currently, latent factors in factor models are explicitly acknowledged as representing postulated causes of the indicator items [6:130, 22:219], but historical disregard for factor causal structuring likely played an obfuscating role during SF-8 development. How else might we explain why pain was not viewed as causing activity limitations rather than pain and limitations correlating solely through their dependence on common-factor causes, or why the word ‘or’ in several items was not flagged for causal infidelity? Confirmatory factor model testing has been available for decades [23], so it is unclear why Ware, Kosinski, Dewey and Gandek [1] did not report test results for their factor models. More recent reports of the failure of SF-8 factor structuring [7, 8] question the common cause style of scale justification provided during the SF-8 development and warrants correction but does not dictate inevitable scale failure, or the outcome of a fusion validity assessment, because diverse entities may meld or fuse to form causally operative scales. Indeed, once scales are created it becomes increasingly difficult to detect scale deficiencies. Our model of the SF-8 mental and physical health scales was consistent with the covariance data and produced appropriately signed though modest effect estimates – and hence raised no red flags. And once a scale becomes available there seems little reason to re-employ the items, but even if someone did the model might fit, as it did with our item-only model, with the only sign of problems being the ineffectiveness of some items.

It takes the simultaneous use of the scale and the items to clearly test a scale. The clear failure of the basic fusion validity model for the SF-8, and the nature of the missed and mistaken causal claims exposed by the fusion model’s failure, highlighted the specific deficiencies of both SF-8 scales. The world seems not to fuse, combine, or meld the SF-8 items into parallel physical or mental health features capable of corresponding real-world causal actions.

We assessed the fusion validity of the two SF-8 scales simultaneously but these scales might have been checked individually had only one scale been causally relevant to the downstream variables. Fusion validity can be checked by re-analysis of any existing SF-8 data set containing at least one variable causally downstream from physical or mental health – whether related to work, play, education, medicine, family, or psychology. Studies that used the SF-8 to assess treatment effectiveness might not include downstream variables permitting fusion validity assessment, but a model containing a treatment causing physical and mental latent common factors having item indicators could at least test the common-factor claim and potentially detect differential item functioning [9, 10, 21]. Studies anticipating future data collection might introduce relevant downstream variables and/or potential item-replacement variables tuned to the research context.

## Summary

Assessing fusion validity of the SF-8 by modeling the scales and indictor items simultaneously revealed severe data inconsistency. Our models collectively demonstrated that some indicator items had many effects, others had few effects, and still others had no detectable effects, and that the scales muddled these item differences. The model containing scales without items (corresponding to typical scale use) matched the scales’ covariances despite misrepresenting the underlying causal structure. This demonstrates that fusion validity assessment can detect scale deficiencies that could otherwise go undetected.

Invalidity of the SF-8 physical and mental scales in the context of Canadian healthcare aides working in long-term care homes raises concern for whether these scales function appropriately in other contexts. The problematic methodology underlying the SF-8 items – inattention to causal structuring among the items, the ‘or’ in items, and the attempt to reduce eight conceptual domains to two scales – suggests the concerns reside in the scales themselves, not in any specific context. This suggests evidence of SF-8 invalidity is likely to accumulate and eventually stimulate concern for how best to recover from extensively used but invalid scales. This assessment may be pessimistic, but prudence recommends at least assessing fusion validity of the SF-8 scales in other settings.

Failure of these seemingly well-established scales raises concern for less-scrutinized scales. Fortunately, fusion validity assessment does not depend on the extent of prior scale development. It requires only two fundamental features: knowing the fixed effects leading from the items to the scale (values available because they are required for calculating scale scores) and postulating at least one variable as causally downstream from the scale.

Invalid scales erode the disciplines and careers they touch, so test your local scales thoroughly and reward, repair, or replace as appropriate. For the SF-8, this will definitely include checking the fusion validity of the SF-8 in different contexts, and will probably include creating new or replacement items.

## Electronic Supplementary Material

Below is the link to the electronic supplementary material.

Supplementary Material 1

## Data Availability

Sufficient data and analytic code to replicate results are provided in the Appendix, and the original data are archived in Health Research Data Repository in the Faculty of Nursing at the University of Alberta. The privacy and ethics approvals covering the TREC data do not permit release of even de-identified individual data. Access to review the original source data may be granted to those with legitimate concerns and meeting pre-specified confidentiality criteria, through a request to the TREC unit data manager (https://trecresearch.ca/about/people) following new ethics approval and consent from the original data providers.

## Footnotes

1 Two other substantive, but inconsequential, decisions were made during early model development. First, the DRAS and DRANS variables (Disruptive Residence Actions of a sexual or nonsexual nature) were modeled as exogenous rather than endogenous variables. These variables were relevant, but we did not intend to model them as causal consequences of physical or mental health, so they were more appropriately modeled as exogenous control variables rather than as ‘unexplained endogenous’ variables. A second matter concerned available TREC data on care aides’ reports of tasks they had left undone during recent work shifts. LH hoped to recode the relevant TREC variables to differentiate between tasks likely left undone due to aides’ physical health limitations and tasks likely left undone due to mental health limitations. However, no recoding provided a statistically converging model.

2 Each SF-8 indicator was specified as having 5% measurement error variance. The reasonableness of this assessment was later checked by re-estimating the item-only and scale-and-item models with half and with double this amount of measurement error variance for each indicator [19]. This produced only trivial changes in results for these models.

3 We ran additional models to confirm specific model features, but none recommended emending the three presented models. We used the item-only model (Table 2) as a base for constructing a model with fixed SF-8 weightings creating the physical and mental health scales as latent variables (paralleling the scales in Fig. 3), but with the scales permitted no effects. This provides modification indices reporting whether the data recommend combining the items into scales having any effects on downstream variables beyond the specific item effects already included in the item-only model. The largest of the eight possible modification indices was close to what might be expected by chance (4.9) with the corresponding expected parameter change value being positive and providing the unreasonable “suggestion” that better health increases tiredness. Thus the already fitting item-only model showed no benefit from including collective effects working through either the SF-8 physical or mental health scale. We also considered the possibility of reciprocal effects among pairs of our endogenous downstream variables. Such effects would likely be underidentified but weaker than the modeled effects, so we estimated a model containing small fixed reciprocal effects in the most probable locations. This made no appreciable difference to the estimates but does argue against reciprocal effects among the downstream variables as providing an explanation for our observations.

## Abbreviations

BE: Bothered by Emotions
BP: Bodily Pain
EN: Energy
LISREL: Linear Structural Relations
PELS: Physical Emotional Limits Social
PELW: Personal Emotional Limits Work
PLA: Physical Limits Activities
PLW: Physical Limits Work
RH: Rated Health
SF-8: Short Form − 8
TREC: Translating Research in Elder Care
χ^2^: Chi-squared
