# Supplementary material for: Evidence pointing toward invalidity of the SF-8 physical and mental scales: a fusion validity assessment
Source: BMC Med Res Methodol. 2024 Nov 11;24:274. doi: 10.1186/s12874-024-02387-z (PMC11552401; doi:10.1186/s12874-024-02387-z)
Supplement: Supplementary file 1 — Supplementary Material 1 [file 12874_2024_2387_MOESM1_ESM.docx]

**APPENDIX A**

This appendix contains LISREL syntax (Joreskog and Sorbom, 1996) for the scale-only and the basic fusion validity model (the scale-and-item model without the direct effects from the items) for the cases having complete information on the modeled variables.

Title SF-8 Scales Only

DA NI=21 NO=3645 MA=CM

CM SY

65.045417

13.538615 72.929394

36.422752 23.968351 49.828730

46.116066 12.169877 23.015827 52.463723

50.471380 17.930121 24.472021 34.771513 52.526577

54.470374 25.771530 28.911143 29.967724 34.519882 75.811445

32.082694 35.726184 25.439655 20.104991 22.326115 29.651682 56.635910

29.667603 44.377577 22.572194 25.593761 28.269802 27.240422 25.425478 60.056967

13.757521 62.176276 20.170131 16.340050 20.533868 24.896042 23.787400 34.683651 60.901453

17.346725 38.977489 14.883896 15.078208 18.482148 18.304657 16.199034 25.615211 30.175915

35.926383

-5.709580 -6.509907 -4.295227 -3.963420 -4.718612 -6.203268 -5.743907 -5.398972

-5.455310 -4.029507 4.252190

-4.678524 -5.994195 -3.759160 -2.935862 -3.583737 -5.646803 -5.835577 -4.866475

-4.869935 -2.994327 1.399480 7.650179

-5.745388 -6.801469 -4.231349 -3.984116 -4.755739 -6.448031 -6.006276 -5.618750

-5.755533 -3.978646 2.147328 1.668469 3.991295

2.024732 3.359480 1.805438 1.435646 1.604148 2.220905 3.605142 2.105768

2.653769 1.625170 -0.750292 -0.917506 -0.860806 1.474012

1.548479 1.721570 0.988735 1.062222 1.169844 2.089138 1.307395 1.439311

1.538937 1.036105 -0.439662 -0.881232 -0.518199 0.188700 1.596405

0.647435 0.837141 0.669601 0.393468 0.399072 0.769665 0.969634 0.655212

0.632644 0.348697 -0.135233 -0.390018 -0.251619 0.179920 0.095205 0.563013

-0.227889 -0.067460 -0.179260 -0.095314 -0.169513 -0.231974 -0.159111 -0.083403

-0.061770 -0.028940 0.007804 0.030702 0.011289 0.005642 -0.014745 -0.004432

0.090332

0.069433 2.404221 0.717727 -0.128249 0.298807 0.176446 1.965587 1.319742

1.703911 1.045768 -0.520071 -0.276065 -0.281977 0.195766 0.164729 -0.037560

0.036716 5.145775

0.386452 0.790912 0.435450 0.042754 0.173854 0.727691 1.093415 0.435411

0.576519 0.113793 -0.108719 -0.299425 -0.096530 0.104114 0.055210 0.052942

-0.009351 0.069689 0.226723

-1.469480 -1.624983 -0.774599 -1.177168 -1.261896 -1.847630 -0.996717 -1.464611

-1.551497 -1.057514 0.421407 0.702990 0.493529 -0.173721 -0.187217 -0.066698

-0.000597 -0.095606 -0.005137 1.710827

-0.591091 -0.813710 -0.379110 -0.395328 -0.642964 -0.657536 -0.442725 -0.692317

-0.749242 -0.532155 0.139008 0.312395 0.155414 -0.036122 -0.086290 -0.024272

0.009887 -0.041527 -0.004429 0.281005 0.449816

LA

SF8Phys SF8Ment

G-Health P-LimitA P-LimitW B-Pain Energy Limit-So EmotProb Limit-Da

Tired Rushed Burnout LookForw

ShortSta Support Sex Age ESL DRAN DRAS /

SE

Tired Rushed Burnout LookForw

SF8Phys SF8Ment ShortSta Support Sex Age ESL DRAN DRAS /

MODEL NY= 4 NX=9 NE=4 NK=9 LY=FU,FI LX=FU,FI BE=FU,FI GA=FU,FI PH=SY,FR PS=SY,FI TE=SY,FI TD=SY,FI

VA 1.0 LY(1,1) LY(2,2) LY(3,3) LY(4,4)

VA 1.0 LX(1,1) LX(2,2) LX(3,3) LX(4,4) LX(5,5) LX(6,6) LX(7,7) LX(8,8) LX(9,9)

FR BE(2,1)

FR BE(3,1) BE(3,2)

FR BE(4,1) BE(4,2) BE(4,3)

FR GA(1,1) GA(1,2) GA(1,3) GA(1,6) GA(1,8)

FR GA(2,1) GA(2,2) GA(2,3) GA(2,4) GA(2,7) GA(2,8) GA(2,9)

FR GA(3,1) GA(3,2) GA(3,3) GA(3,4) GA(3,7) GA(3,8)

FR GA(4,1) GA(4,2) GA(4,4) GA(4,5) GA(4,7) GA(4,8) GA(4,9)

FR PS(1,1) PS(2,2) PS(3,3) PS(4,4)

VA 0.085043809 TE(1,1) ! 2% of 4.25219046 Tired AM

VA 0.535512502 TE(2,2) ! 7% of 7.6501786 Rushed

VA 0.159651819 TE(3,3) ! 4% of 3.99129549 Burnout

VA 0.058960467 TE(4,4) ! 4% of 1.47401169 Look Forward

! TD(1,1) TD(2,2) for SF-8 physical and mental scales left set at zero

VA 0.079820255 TD(3,3) ! 5% of 1.5964051 Enough Staff

VA 0.022520505 TD(4,4) ! 4% of 0.56301263 Supportive

VA 0.002709954 TD(5,5) ! 3% of 0.09033181 Sex

VA 0.205831013 TD(6,6) ! 4% of 5.14577533 Age

VA 0.045344692 TD(7,7) ! 20% of 0.22672346 ESL

VA 0.085541341 TD(8,8) ! 5% of 1.71082683 DRAN

VA 0.02249078 TD(9,9) ! 5% of 0.44981561 DRAS

OU ML ND=3 WP SE TV RS MI SS EF

=============================================================

Title SF-8 SCALE-and-ITEM model, with the indicator items having no direct effects.

! This is the basic fusion validity model.

DA NI=21 NO=3645 MA=CM

CM SY

65.045417

13.538615 72.929394

36.422752 23.968351 49.828730

46.116066 12.169877 23.015827 52.463723

50.471380 17.930121 24.472021 34.771513 52.526577

54.470374 25.771530 28.911143 29.967724 34.519882 75.811445

32.082694 35.726184 25.439655 20.104991 22.326115 29.651682 56.635910

29.667603 44.377577 22.572194 25.593761 28.269802 27.240422 25.425478 60.056967

13.757521 62.176276 20.170131 16.340050 20.533868 24.896042 23.787400 34.683651

60.901453

17.346725 38.977489 14.883896 15.078208 18.482148 18.304657 16.199034 25.615211

30.175915 35.926383

-5.709580 -6.509907 -4.295227 -3.963420 -4.718612 -6.203268 -5.743907 -5.398972

-5.455310 -4.029507 4.252190

-4.678524 -5.994195 -3.759160 -2.935862 -3.583737 -5.646803 -5.835577 -4.866475

-4.869935 -2.994327 1.399480 7.650179

-5.745388 -6.801469 -4.231349 -3.984116 -4.755739 -6.448031 -6.006276 -5.618750

-5.755533 -3.978646 2.147328 1.668469 3.991295

2.024732 3.359480 1.805438 1.435646 1.604148 2.220905 3.605142 2.105768

2.653769 1.625170 -0.750292 -0.917506 -0.860806 1.474012

1.548479 1.721570 0.988735 1.062222 1.169844 2.089138 1.307395 1.439311

1.538937 1.036105 -0.439662 -0.881232 -0.518199 0.188700 1.596405

0.647435 0.837141 0.669601 0.393468 0.399072 0.769665 0.969634 0.655212

0.632644 0.348697 -0.135233 -0.390018 -0.251619 0.179920 0.095205 0.563013

-0.227889 -0.067460 -0.179260 -0.095314 -0.169513 -0.231974 -0.159111 -0.083403

-0.061770 -0.028940 0.007804 0.030702 0.011289 0.005642 -0.014745 -0.004432

0.090332

0.069433 2.404221 0.717727 -0.128249 0.298807 0.176446 1.965587 1.319742

1.703911 1.045768 -0.520071 -0.276065 -0.281977 0.195766 0.164729 -0.037560

0.036716 5.145775

0.386452 0.790912 0.435450 0.042754 0.173854 0.727691 1.093415 0.435411

0.576519 0.113793 -0.108719 -0.299425 -0.096530 0.104114 0.055210 0.052942

-0.009351 0.069689 0.226723

-1.469480 -1.624983 -0.774599 -1.177168 -1.261896 -1.847630 -0.996717 -1.464611

-1.551497 -1.057514 0.421407 0.702990 0.493529 -0.173721 -0.187217 -0.066698

-0.000597 -0.095606 -0.005137 1.710827

-0.591091 -0.813710 -0.379110 -0.395328 -0.642964 -0.657536 -0.442725 -0.692317

-0.749242 -0.532155 0.139008 0.312395 0.155414 -0.036122 -0.086290 -0.024272

0.009887 -0.041527 -0.004429 0.281005 0.449816

LA

SF8Phys SF8Ment G-Health P-LimitA P-LimitW B-Pain Energy Limit-So

EmotProb Limit-Da Tired Rushed Burnout LookForw ShortSta Support Sex Age

ESL DRAN DRAS /

SE

G-Health P-LimitA P-LimitW B-Pain Energy Limit-So EmotProb Limit-Da

Tired Rushed Burnout LookForw ShortSta Support Sex Age ESL DRAN DRAS /

MODEL NY= 12 NX=7 NE=14 NK=15 LY=FU,FI LX=FU,FI BE=FU,FI GA=FU,FI PH=SY,FR PS=SY,FI TE=SY,FI TD=SY,FI

VA 1.0 LY(1,1) LY(2,2) LY(3,3) LY(4,4) LY(5,5) LY(6,6) LY(7,7) LY(8,8) LY(9,11) LY(10,12)

VA 1.0 LY(11,13) LY(12,14)

VA 1.0 LX(1,9) LX(2,10) LX(3,11) LX(4,12) LX(5,13) LX(6,14) LX(7,15)

VA 0.2xxxx BE(9,1) !See Ware, et al 2001 for the precise SF-8 item weightings.

VA 0.3xxxx BE(9,2) !See Ware, et al 2001 for the precise SF-8 item weightings.

VA 0.4xxxx BE(9,3) !See Ware, et al 2001 for the precise SF-8 item weightings.

VA 0.3xxxx BE(9,4) !See Ware, et al 2001 for the precise SF-8 item weightings.

VA 0.1xxxx BE(9,5) !See Ware, et al 2001 for the precise SF-8 item weightings.

VA 0.0xxxx BE(9,6) !See Ware, et al 2001 for the precise SF-8 item weightings.

VA -0.2xxxx BE(9,7) !See Ware, et al 2001 for the precise SF-8 item weightings.

VA 0.0xxxx BE(9,8) !See Ware, et al 2001 for the precise SF-8 item weightings.

VA 0.0xxxx BE(10,1) !See Ware, et al 2001 for the precise SF-8 item weightings.

VA -0.1xxxx BE(10,2) !See Ware, et al 2001 for the precise SF-8 item weightings.

VA -0.1xxxx BE(10,3) !See Ware, et al 2001 for the precise SF-8 item weightings.

VA -0.0xxxx BE(10,4) !See Ware, et al 2001 for the precise SF-8 item weightings.

VA 0.2xxxx BE(10,5) !See Ware, et al 2001 for the precise SF-8 item weightings.

VA 0.2xxxx BE(10,6) !See Ware, et al 2001 for the precise SF-8 item weightings.

VA 0.7xxxx BE(10,7) !See Ware, et al 2001 for the precise SF-8 item weightings.

VA 0.3xxxx BE(10,8) !See Ware, et al 2001 for the precise SF-8 item weightings.

! The BE matrix PERMITTING scale effects (eta 9 and eta10), and downstream effects.

FR BE(11,9) BE(11,10)

FR BE(12,9) BE(12,10) BE(12,11)

FR BE(13,9) BE(13,10) BE(13,11) BE(13,12)

FR BE(14,9) BE(14,10) BE(14,11) BE(14,12) BE(14,13)

VA 1.0 GA(1,1) GA(2,2) GA(3,3) GA(4,4) GA(5,5) GA(6,6) GA(7,7) GA(8,8)

! The exogenous variable direct effects, also documenting the seven direct item effects added

! to the basic fusion validity model to obtain the scale-and-item model in Figure 3.

FR GA(11,9) GA(11,12) GA(11,14)

!ADDED LATER GA(11,4) GA(11,5)

FR GA(12,9) GA(12,10) GA(12,13) GA(12,14) GA(12,15)

!ADDED LATER GA(12,5)

FR GA(13,9)GA(13,10) GA(13,13) GA(13,14)

!ADDED LATER GA(13,4) GA(13,5)

FR GA(14,10) GA(14,11) GA(14,13) GA(14,14) GA(14,15)

!ADDED LATER GA(14,4) GA(14,5)

ST 50. PH(3,3) PH(8,8)

ST 60. PH(1,1) PH(5,5)

ST 70. PH(2,2) PH(4,4) PH(6,6) PH(7,7)

ST 1.4 PH(9,9)

ST 0.5 PH(10,10)

ST 0.09 PH(11,11)

ST 4.8 PH(12,12)

ST 0.2 PH(13,13)

ST 1.6 PH(14,14)

ST 0.4 PH(15,15)

! The SF-8 items are given 5% measurement error variance, with later sensitivity analyses

! using 2.5%, and 10% finding no substantial change in effect estimates.

VA 2.9942585 PS(1,1) ! 5% of 59.88517

VA 3.767599462 PS(2,2) ! 5% of 75.35198924

VA 2.442037929 PS(3,3) ! 5% of 48.84075858

VA 4.275139117 PS(4,4) ! 5% of 85.50278235

VA 3.036700518 PS(5,5) ! 5% of 60.73401036

VA 3.357844951 PS(6,6) ! 5% of 67.15689902

VA 4.144546246 PS(7,7) ! 5% of 82.89092492

VA 2.143147933 PS(8,8) ! 5% of 42.86295866

VA 0.0 PS(9,9)

VA 0.0 PS(10,10)

FR PS(11,11)

FR PS(12,12)

FR PS(13,13)

FR PS(14,14)

! TE(1,1) to TE(8,8) are zero because SF-8 measurement errors are modeled in PS

VA 0.0 TE(1,1) TE(2,2) TE(3,3) TE(4,4) TE(5,5) TE(6,6) TE(7,7) TE(8,8)

VA 0.085043809 TE(9,9) ! 2% of 4.25219046 Tired AM

VA 0.535512502 TE(10,10) ! 7% of 7.6501786 Rushed

VA 0.159651819 TE(11,11) ! 4% of 3.99129549 Burnout

VA 0.058960467 TE(12,12) ! 4% of 1.47401169 LookForward

VA 0.079820255 TD(1,1) ! 5% of 1.5964051

VA 0.022520505 TD(2,2) ! 4% of 0.56301263

VA 0.002709954 TD(3,3) ! 3% of 0.09033181

VA 0.205831013 TD(4,4) ! 4% of 5.14577533

VA 0.045344692 TD(5,5) ! 20% of 0.22672346

VA 0.085541341 TD(6,6) ! 5% of 1.71082683

VA 0.02249078 TD(7,7) ! 5% of 0.44981561

OU ML ND=3 WP SE TV RS MI SS EF AD=OFF IT=300
